# Twist-Bent Bonds Revisited: Adiabatic Ionization Potentials Demystify Enhanced Reactivity

**DOI:** 10.1021/acsomega.2c05074

**Published:** 2022-10-11

**Authors:** Abhik Ghosh, Jeanet Conradie

**Affiliations:** † Department of Chemistry, University of Tromsø, N-9037 Tromsø, Norway; ‡ Department of Chemistry, University of the Free State, 9300 Bloemfontein, Republic of South Africa

## Abstract

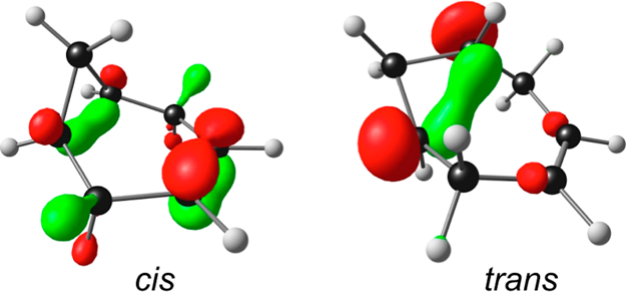

Explicit calculations of vertical and adiabatic ionization potentials of cyclopropane derivatives with modern DFT methods have underscored the possibility of unusually large reorganization energies (defined as the difference between vertical and adiabatic ionization potentials) of 0.5–1.0 eV for several compounds. Such is the case for ionization of the twist-bent σ-bond of *trans*-bicyclo[4.1.0]hept-3-ene (*trans*-3-norcarene), for which B3LYP*-D3 calculations predict an adiabatic IP of 7.92 eV. The corresponding value for the *cis*-norcarene is 8.34 eV. The significantly lower adiabatic IP provides an attractive explanation for the higher reactivity of the *trans* compound under oxidative conditions. Large reorganization energies are also found for the ionization of cyclopropane, bicyclo[1.1.0]butane, and bicyclo[2.1.0]pentane. In sharp contrast, an exceptionally small reorganization energy is associated with the ionization of tricyclo[1.1.1.0]pentane ([1.1.1]propellane).

## Introduction

**Scheme 1:**
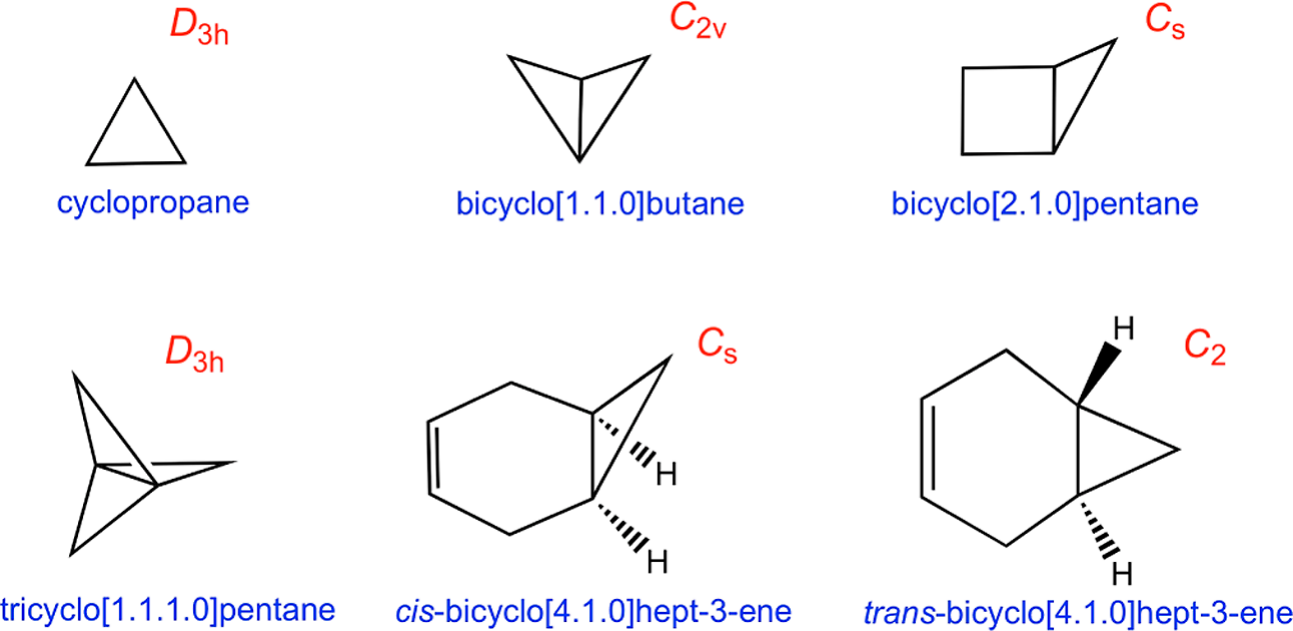
Cyclopropane Derivatives Studied in This Work

The middle decades of the 20th century (1925–1975) were arguably the golden age of physical organic chemistry,^1−3^ and the concept of strain,^4−6^ that is, deviations of bond distances and angles from their normative values, played a key role in the development of the subject. This period also saw the emergence of gas-phase photoelectron spectroscopy (PES) applied to a plethora of organic molecules, which confirmed key predictions from molecular orbital calculations.^7,8^ Gas-phase PES proved particularly insightful for strained hydrocarbons and especially for cyclopropane derivatives.^9,10^ The theoretical tools available at the time, however, could only provide plausible, rather than conclusive, assignments of the spectra. Herein, accordingly, we have revisited the lowest ionization potentials (IPs) of selected cyclopropane derivatives with modern DFT calculations with a view to reliably characterizing the lowest ionized states (Scheme 1).^11−17^

The photoelectron spectra of cyclopropane derivatives are chock-full of subtleties and mysteries.^9,10^ Thus, while cyclopropane exhibits an experimental IP of 9.86 eV,^18−21^ the corresponding value for the seemingly far more strained tricyclo[1.1.1.0]pentane (commonly known as [1.1.1]propellane) is marginally different, 9.74 eV.^22^ In contrast, the experimental vertical IP of bicyclo[1.1.0]butane is significantly lower, 8.70 eV,^23^ as is that of the potentially less strained *cis*-bicyclo[4.1.0]hept-3-ene (*cis*-3-norcarene), 9.05 eV.^24^ As it happens, *trans*-bicyclo[4.1.0]hept-3-ene (*trans*-3-norcarene) exhibits an essentially identical vertical IP, 9.00 eV.^24^ In other words, our intuitive ideas about angle strain are a poor guide to the PES data, which brings us to the subject of bent bonds and, in particular, twist-bent bonds.^25−27^

**Scheme 2:**
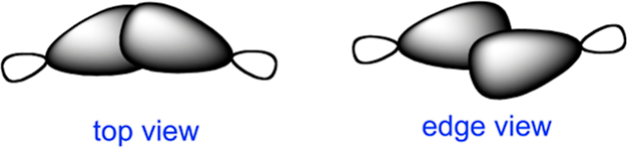
Top and Edge Views of Twist-Bent Bonds

Cyclopropane provides arguably the best-known example of bent bonds, in which the interorbital angle for C–C bonds (104–105°) is much wider than the internuclear bond angle (60°).^28,29^ Accordingly, the path of maximum electron density between two carbons curves outside the CCC equilateral triangle approximately in the form of a banana, which has led to the moniker “banana bonds” for the CC bonds in cyclopropane. Over 50 years ago,^30,31^ Gassman recognized that the ring-fusion bond in strained *trans*-fused cyclopropane derivatives such as norcarane exhibits an altogether different topology. Seen from the cyclopropane carbon atom across the ring-fusion bond, the latter is found to exhibit a sigmoidal topology,^30,31^ as depicted in Scheme 2.

Gassman described the two topologies as “symmetrically bent” and “twist-bent”,^30,31^ even though the latter also exhibits approximate *C*_2_ local symmetry in terms of Mislow’s terminology on local symmetry groups.^32^ Some may choose to analogize a twist-bent bond to an incipient conrotatory ring-opening of a cyclopropane.^33^ Working primarily with *trans*-3-norcarene derivatives, Gassman and co-workers showed that the twist-bent bond is significantly more reactive than the normal ring-fusion bond in *cis*-3-norcarene.^34−36^

How then can we understand that *cis*- and *trans*-3-norcarene exhibit essentially identical vertical IPs?^24^ Early DFT calculations, published posthumously for Gassman, showed that although the lowest vertical IP corresponds to the twist-bent ring-fusion bond for the *trans* isomer, it corresponds to the C–C π-bond (i.e., a part of the C–C double bond) at the 3-position in the *cis* isomer.^24^ The present B3LYP* calculations confirm this conclusion but also shed additional light on the subject.

## Results and Discussion

**Figure 1:**
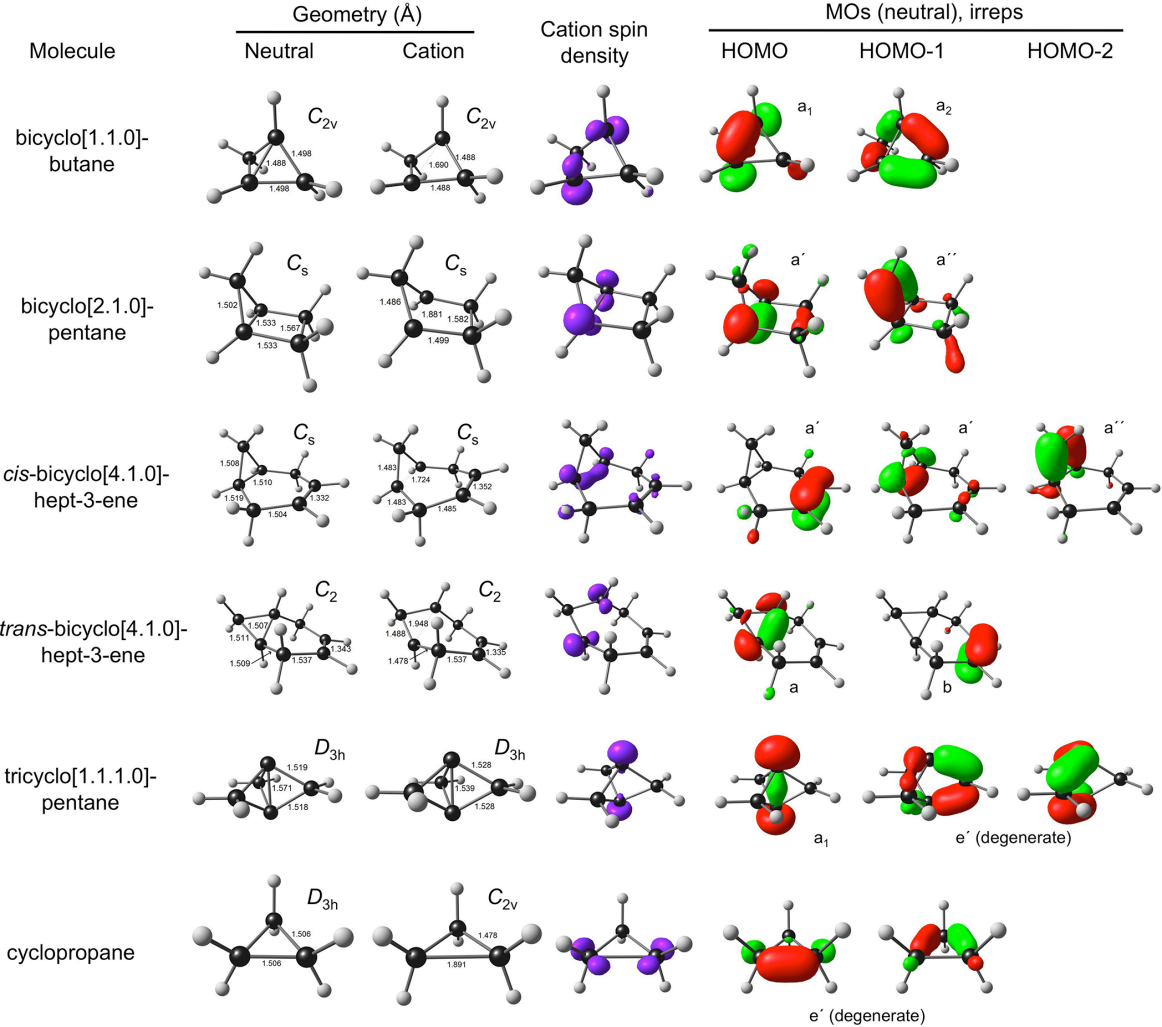
Selected B3LYP*-D3/STO-QZ4P results from left to right: selected optimized bond distances (Å) for the neutral and cationic states, spin density of the cation, and selected highest occupied MOs (in general, HOMO and HOMO – 1).

In this study, we have examined six cyclopropane derivatives and their ionized states with B3LYP*^37,38^-D3^39^/ZORA^40^-STO-QZ4P calculations and the ADF 2019 program.^41^Table 1 lists calculated vertical and adiabatic IPs and also the corresponding experimental values, when available. Figure 1 depicts the optimized geometries of the neutral and cationic states, the spin density profiles of the optimized cations, and 2–3 HOMOs of the neutral molecules. The calculated IPs generally agree with experimental values to within 0.2–0.3 eV, while differences in IPs among different molecules are in even better agreement with experiment. Overall, the calculations do a good job of reproducing the somewhat perplexing trends in IPs mentioned above.

**Table 1:** DFT Calculations of Vertical (IP_v1_ and IP_v2_) and Adiabatic (IP_a_) Ionization Potentials (eV) for Selected Cyclopropane Derivatives, along with Available Experimental Values

	calculated^a^				
molecule	IP_a_	IP_v1_	IP_v2_	experimental	ref
cyclopropane	9.66			9.86 (IP_a_)	(18)
bicyclo[1.1.0]butane	8.55	9.15		8.70 (IP_v1_)	(23)
bicyclo[2.1.0]pentane	8.34	9.30	9.93	(IP_v1_: 8.6)^b^	(42)
tricyclo[1.1.1.0]pentane	9.55	9.60		9.74 (IP_v1_)	(22)
*cis*-bicyclo[4.1.0]hept-3-ene	8.34	8.63	9.75	9.05 (IP_v1_), 9.85 (IP_v2_)	(24)
*trans*-bicyclo[4.1.0]hept-3-ene	7.92	8.67	9.00	9.00 (IP_v1_), 9.30 (IP_v2_)	(24)

a Vertical IPs were not calculated when the neutral MOs in question are degenerate by symmetry.

b This value is derived from PES measurements on bicyclo[2.1.0]pent-2-ene.

A key new insight from this work is that large reorganization effects may be involved for ionization of many, but not all, cyclopropane derivatives. In other words, the neutral and cationic states may differ significantly, even dramatically, in terms of their geometries. Understandably, the adiabatic IPs in such cases may be dramatically lower than the vertical IPs. Thus, upon ionization, one of the ionized bonds of cyclopropane expands from 1.506 to 1.891 Å (Figure 1), that is, by almost 0.4 Å, consistent with a 0.75 eV difference between experimental vertical and adiabatic IPs, which have been reported as 9.86^18^ and 10.60^19^ eV, respectively. Large reorganization effects are also observed for bicyclo[1.1.0]butane and bicyclo[2.1.0]pentane (Table 1). Thus, the calculated adiabatic IP of bicyclo[1.1.0]butane (8.55 eV) is about 0.6 eV lower than the vertical IP (9.15 eV). Likewise, the calculated adiabatic IP of bicyclo[2.1.0]pentane (8.34 eV) is about 0.96 eV lower than the vertical IP (9.30 eV), which corresponds to a reorganization energy of almost 1 eV! It is worth noting that the relevant experimental value (8.6 eV) quoted in Table 1 is not for the same compound but for the structurally related bicyclo[2.1.0]pent-2-ene.^42^ As in the case of cyclopropane, the large reorganization energies appear to be clearly related to large geometrical changes accompanying ionization. In sharp contrast, only a very small reorganization energy is associated with the ionization of tricyclo[1.1.1.0]pentane ([1.1.1]propellane); the optimized DFT geometry, consistent with our rationale, reveals only a small shrinkage of the central C–C bond upon ionization (Figure 1), a consequence of the remarkable polycyclic structure.^22^

**Figure 2:**
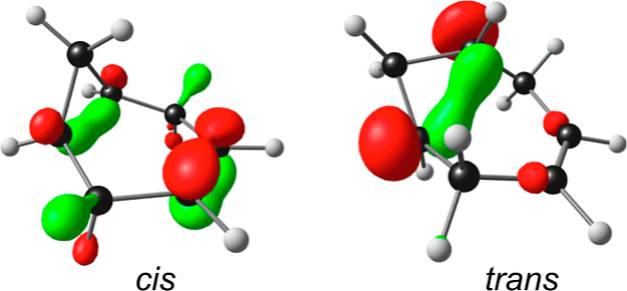
SOMOs of *cis*- and *trans*-bicyclo[4.1.0]hept-3-ene cations.

In the case of the 3-norcarenes, the adiabatically ionized states of both the *cis* and *trans* isomers are similar in that both involve ionization of the ring-fusion bond, unlike in the vertically ionized states, where the ionization occurs from different orbitals, as mentioned above. This difference may be seen from the singly occupied MOs (SOMOs) of the two cations (Figure 2) and from the spin density plots included as part of Figure 1. However, the two cations differ in important ways. The adiabatic IP of *trans*-3-norcarene is the lowest among the cyclopropane derivatives examined in this study, a good 0.4 eV lower than that of *cis*-3-norcarene, which appears to dovetail with a significant difference in the spin density profiles of the two cations. Thus, most of the spin density in the *trans*-3-norcarene cation has drained out of the internuclear region of the ionized bond (in much the same way as in the majority of the other compounds studied), whereas the spin density in the *cis*-3-norcarene cation is still substantially concentrated in the internuclear region. We suggest that the difference in adiabatic IP and topological differences in the cation spin density profiles go a long way toward explaining the higher reactivity of *trans*-3-norcarene under oxidative conditions.^34−36^

## Conclusions

Revisiting the bent bonds in cyclopropane derivatives including *cis*- and *trans*-bicyclo[4.1.0]hept-3-ene has led to a more detailed appreciation of such bonds. While confirming that twist-bent bonds are easier to ionize than regular “banana” bonds in related compounds (in terms of vertical ionization potentials), the present calculations also reveal a significantly larger reorganization energy associated with ionization of the former, which, in our view, is likely to underpin the higher reactivity of twist-bent bonds.

## Supporting Information Available

Optimized B3LYP-D3/ZORA-STO-TZ2P coordinates (PDF)
