# Supplementary material for: Twist-Bent Bonds Revisited: Adiabatic Ionization Potentials Demystify Enhanced Reactivity
Source: ACS Omega. 2022 Oct 11;7(42):37917–21. doi: 10.1021/acsomega.2c05074 (PMC9608396; doi:10.1021/acsomega.2c05074)
Supplement: Supplementary file 1 — ao2c05074_si_001.pdf [file ao2c05074_si_001.pdf]

Twist-Bent Bonds Revisited: Adiabatic Ionization Potentials Demystify Enhanced Reactivity

Abhik Ghosh*[,a] and Jeanet Conradie*[,a,b]

[a]Department of Chemistry, University of Tromsø, N-9037 Tromsø, Norway;

[b]Department of Chemistry, University of the Free State, 9300 Bloemfontein, Republic of South Africa.

Supporting information

Optimized Cartesian coordinates (Å)

All compounds were optimized the ZORA Hamiltonian, all electron ZORA/QZ4P basis set, B3LYP* functional.

Table of Contents

1. Bicyclo[1.1.0]butane, C_{2v}, q = 0, S = 0 2
2. Bicyclo[2.1.0]pentane, C_s, q = 0, S = 0 2
3. *Cis*-3-norcarene, C_s, q = 0, S = 0 2
4. *Trans*-3-norcarene, C_2, q = 0, S = 0 3
5. Cyclopropane, D_{3h}, q = 0, S = 0 3
6. propallane, C_{3v}, q = 0, S = 0 3
7. Bicyclo[1.1.0]butane, C_{2v}, q = 1, S = ½ 4
8. Bicyclo[2.1.0]pentane, C_s, q = 1, S = ½ 4
9. *Cis*-3-norcarene, C_s, q = 1, S = ½ 4
10. *Trans*-3-norcarene, C_2, q = 1, S = ½ 5
11. [1.1.1]Propellane, C_{3v}, q = 1, S = ½ 5
12. Cyclopropane, C_{2v}, q = 1, S = ½ 5

1. Bicyclo[1.1.0]butane, C_{2v}, q = 0, *S* = 0

C	0.000000000	1.136184000	0.314719000
C	0.000000000	-1.136184000	0.314719000
C	0.744125000	0.000000000	-0.317543000
C	-0.744125000	0.000000000	-0.317543000
H	0.000000000	1.234236000	1.400436000
H	0.000000000	2.080892000	-0.219248000
H	0.000000000	-1.234236000	1.400436000
H	0.000000000	-2.080892000	-0.219248000
H	1.428505000	0.000000000	-1.149144000
H	-1.428505000	0.000000000	-1.149144000

2. Bicyclo[2.1.0]pentane, C_s, q = 0, *S* = 0

C	1.029681000	0.107987000	0.783282000
C	1.029681000	0.107987000	-0.783282000
C	-0.416014000	-0.402578000	0.766383000
C	-0.416014000	-0.402578000	-0.766383000
C	-1.293702000	0.544951000	0.000000000
H	1.188637000	1.069358000	1.279079000
H	1.188637000	1.069358000	-1.279079000
H	1.734683000	-0.615609000	1.191660000
H	1.734683000	-0.615609000	-1.191660000
H	-0.832082000	-1.135634000	1.444947000
H	-0.832082000	-1.135634000	-1.444947000
H	-1.077684000	1.610307000	0.000000000
H	-2.349774000	0.304349000	0.000000000

3. *Cis*-3-norcarene, C_s, q = 0, *S* = 0

C	0.855700000	-0.624206000	-0.755166000
C	0.855700000	-0.624206000	0.755166000
C	1.697259000	0.374324000	0.000000000
C	-0.376363000	-0.159354000	1.511900000
C	-0.376363000	-0.159354000	-1.511900000
C	-1.363061000	0.598091000	0.666006000
C	-1.363061000	0.598091000	-0.666006000
H	1.366062000	1.406323000	0.000000000
H	1.397594000	-1.430472000	1.235990000
H	1.397594000	-1.430472000	-1.235990000
H	2.772015000	0.247292000	0.000000000
H	-0.081027000	0.454081000	2.371564000
H	-0.081027000	0.454081000	-2.371564000
H	-0.882512000	-1.034307000	1.940086000
H	-0.882512000	-1.034307000	-1.940086000
H	-2.126268000	1.159941000	1.195213000

H	-2.126268000	1.159941000	-1.195213000

4. *Trans*-3-norcarene, C_2, q = 0, *S* = 0

C	0.000000000	0.000000000	-2.093072000
C	0.625620000	0.419997000	-0.783582000
C	0.668964000	-0.061075000	1.550334000
C	1.553988000	-0.085735000	0.293422000
C	-0.625620000	-0.419997000	-0.783582000
C	-0.668964000	0.061075000	1.550334000
C	-1.553988000	0.085735000	0.293422000
H	0.384407000	1.463789000	-0.592883000
H	0.402662000	-0.813691000	-2.684713000
H	1.179690000	-0.106959000	2.507410000
H	1.922930000	-1.099067000	0.099366000
H	2.435079000	0.545465000	0.450234000
H	-0.384407000	-1.463789000	-0.592883000
H	-0.402662000	0.813691000	-2.684713000
H	-1.179690000	0.106959000	2.507410000
H	-1.922930000	1.099067000	0.099366000
H	-2.435079000	-0.545465000	0.450234000

5. Cyclopropane, D_{3h}, q = 0, *S* = 0

C	-0.434607000	-0.752762000	0.000000000
C	-0.434607000	0.752762000	0.000000000
C	0.869214000	0.000000000	0.000000000
H	-0.728562000	1.261906000	0.908101000
H	-0.728562000	-1.261906000	-0.908101000
H	1.457123000	0.000000000	-0.908101000
H	1.457123000	0.000000000	0.908101000
H	-0.728562000	1.261906000	-0.908101000
H	-0.728562000	-1.261906000	0.908101000

6. [1.1.1]propallane, C_{3v}, q = 0, *S* = 0

C	0.000000000	0.000000000	0.800162000
C	0.000000000	0.000000000	-0.770977000
C	1.299363000	0.000000000	0.015201000
C	-0.649681000	1.125281000	0.015201000
C	-0.649681000	-1.125281000	0.015201000
H	1.880720000	0.914192000	0.015189000
H	1.880720000	-0.914192000	0.015189000
H	-0.148647000	2.085847000	0.015189000
H	-0.148647000	-2.085847000	0.015189000
H	-1.732073000	1.171656000	0.015189000
H	-1.732073000	-1.171656000	0.015189000

7. Bicyclo[1.1.0]butane, C_{2v}, q = 1, S = ½

C	0.000000000	1.131370000	0.254072000
C	0.000000000	-1.131370000	0.254072000
C	0.845055000	0.000000000	-0.213735000
C	-0.845055000	0.000000000	-0.213735000
H	0.000000000	1.259895000	1.346516000
H	0.000000000	2.074007000	-0.284759000
H	0.000000000	-1.259895000	1.346516000
H	0.000000000	-2.074007000	-0.284759000
H	1.505927000	0.000000000	-1.072874000
H	-1.505927000	0.000000000	-1.072874000

8. Bicyclo[2.1.0]pentane, C_s, q = 1, S = ½

C	1.020920000	0.088307000	0.790762000
C	1.020920000	0.088307000	-0.790762000
C	-0.429111000	-0.261362000	0.940385000
C	-0.429111000	-0.261362000	-0.940385000
C	-1.307212000	0.481511000	0.000000000
H	1.262587000	1.069323000	1.213302000
H	1.262587000	1.069323000	-1.213302000
H	1.686566000	-0.657552000	1.216376000
H	1.686566000	-0.657552000	-1.216376000
H	-0.795628000	-1.107703000	1.511860000
H	-0.795628000	-1.107703000	-1.511860000
H	-1.141214000	1.563888000	0.000000000
H	-2.353591000	0.199231000	0.000000000

9. *Cis*-3-norcarene, C_s, q = 1, S = ½

C	0.907047000	-0.557791000	0.862118000
C	0.907047000	-0.557791000	-0.862118000
C	1.666738000	0.379748000	0.000000000
C	-0.386539000	-0.184397000	1.483654000
C	-0.386539000	-0.184397000	-1.483654000
C	-1.315429000	0.646817000	0.675974000
C	-1.315429000	0.646817000	-0.675974000
H	1.299365000	1.404439000	0.000000000
H	1.423674000	-1.421544000	1.260099000
H	1.423674000	-1.421544000	-1.260099000
H	2.743740000	0.256085000	0.000000000
H	-0.144765000	0.343836000	2.427940000
H	-0.144765000	0.343836000	-2.427940000
H	-0.922623000	-1.074081000	1.851305000
H	-0.922623000	-1.074081000	-1.851305000
H	-2.074556000	1.204765000	1.210988000
H	-2.074556000	1.204765000	-1.210988000

10. *Trans*-3-norcarene, C_2, q = 1, *S* = ½

C	0.000000000	0.000000000	-1.945188000
C	0.665828000	-0.046364000	1.521567000
C	0.783419000	0.578983000	-0.820864000
C	1.546945000	-0.075062000	0.262563000
C	-0.665828000	0.046364000	1.521567000
C	-0.783419000	-0.578983000	-0.820864000
C	-1.546945000	0.075062000	0.262563000
H	0.485796000	-0.770027000	-2.539445000
H	0.533270000	1.615871000	-0.612242000
H	1.199239000	-0.069401000	2.464009000
H	1.889273000	-1.084181000	0.022313000
H	2.433715000	0.538105000	0.467744000
H	-0.485796000	0.770027000	-2.539445000
H	-0.533270000	-1.615871000	-0.612242000
H	-1.199239000	0.069401000	2.464009000
H	-1.889273000	1.084181000	0.022313000
H	-2.433715000	-0.538105000	0.467744000

11. [1.1.1]propallane, C_{3v}, q = 1, *S* = ½

C	0.000000000	0.000000000	0.784013000
C	0.000000000	0.000000000	-0.754582000
C	1.320433000	0.000000000	0.015199000
C	-0.660217000	1.143529000	0.015199000
C	-0.660217000	-1.143529000	0.015199000
H	1.886602000	0.923602000	0.015150000
H	1.886602000	-0.923602000	0.015150000
H	-0.143438000	2.095646000	0.015150000
H	-0.143438000	-2.095646000	0.015150000
H	-1.743164000	1.172044000	0.015150000
H	-1.743164000	-1.172044000	0.015150000

12. Cyclopropane, C_{2v}, q = 1, *S* = ½

C	0.000000000	0.945494000	-0.319152000
C	0.000000000	-0.945494000	-0.319152000
C	0.000000000	0.000000000	0.817457000
H	0.930015000	-1.308691000	-0.740686000
H	-0.930015000	1.308691000	-0.740686000
H	-0.924849000	0.000000000	1.391795000
H	0.924849000	0.000000000	1.391795000
H	-0.930015000	-1.308691000	-0.740686000
H	0.930015000	1.308691000	-0.740686000
